# Clinical symptoms and performance on the continuous performance test in children with attention deficit hyperactivity disorder between subtypes: a natural follow-up study for 6 months

**DOI:** 10.1186/1471-244X-11-65

**Published:** 2011-04-19

**Authors:** Liang-Jen Wang, Yu-Shu Huang, Yuan-Lin Chiang, Chen-Cheng Hsiao, Zong-Yi Shang, Chih-Ken Chen

**Affiliations:** 1 Department of Psychiatry, Chang Gung Memorial Hospital at Keelung, Keelung, Taiwan; 2 Master of Public Health, College of Public Health, National Taiwan University, Taipei, Taiwan; 3 Chang Gung University School of Medicine, Taoyuan, Taiwan; 4 Department of Psychiatry, Chang Gung Memorial Hospital at Linko, Taoyuan, Taiwan; 5 Division of Mental Health & Drug Abuse Research, National Health Research Institutes, Miaoli, Taiwan

**Keywords:** ADHD, subtype, aggressive, Continuous Performance Test, clinical symptoms

## Abstract

**Background:**

The aims of this study were to determine the time course of improvements in attention deficit hyperactivity disorder (ADHD) clinical symptoms and neurocognitive function in a realistic clinical setting, and the differences in ADHD symptom improvement using different classifications of ADHD subtypes.

**Methods:**

The Child Behavior Checklist (CBCL) was completed by parents of ADHD children at the initial visit. The computerized Continuous Performance Test (CPT), Swanson, Nolan, and Pelham, and Version IV Scale for ADHD (SNAP-IV), and ADHD Rating Scale (ADHD-RS) were performed at baseline, one month, three months, and six months later, respectively. Patient care including drug therapy was performed at the discretion of the psychiatrist. The ADHD patients were divided into DSM-IV subtypes (Inattentive, Hyperactive-impulsive and Combined type), and were additionally categorized into aggressive and non-aggressive subtypes by aggression scale in CBCL for comparisons.

**Results:**

There were 50 ADHD patients with a mean age of 7.84 ± 1.64 years; 15 of them were inattentive type, 11 were hyperactive-impulsive type, and 24 were combined type. In addition, 28 of the ADHD patients were grouped into aggressive and 22 into non-aggressive subtypes. There were significant improvements in clinical symptoms of hyperactivity and inattention, and impulsivity performance in CPT during the 6-month treatment. The clinical hyperactive symptoms were significantly different between ADHD patients sub-grouping both by DSM-IV and aggression. Non-aggressive patients had significantly greater changes in distraction and impulsivity performances in CPT from baseline to month 6 than aggressive patients.

**Conclusions:**

We found that ADHD symptoms, which included impulsive performances in CPT and clinical inattention and hyperactivity dimensions, had improved significantly over 6 months under pragmatic treatments. The non-aggressive ADHD patients might have a higher potential for improving in CPT performance than aggressive ones. However, it warrant further investigation whether the different classifications of ADHD patients could be valid for predicting the improvements in ADHD patients' clinical symptoms and neurocognitive performance.

## Background

Attention deficit hyperactivity disorder (ADHD), which occurs in 3% to 10% of school-age children, is one of the most common child and adolescent psychiatric disorders [1,2]. ADHD in children has been shown to have a significantly negative influence on global aspects of academic performance, family function, and interpersonal relationships [3,4]. Several studies have demonstrated that ADHD is associated with cognitive impairments on neuropsychological tests [5,6]. Because of the heterogeneity of symptoms in ADHD, the history of classifying ADHD is rife with debate.

Early concerns about ADHD classification were raised over whether it is a broad sense of conduct disorder or a distinct externalizing category [7]. Some studies have emphasized the importance of distinguishing between children with ADHD alone and those with a combination of ADHD symptoms and aggression [8]. Aggression appeared to be a useful means of subtyping ADHD children with respect to behavior, cognitive performance, family function and later outcome [9,10]. Different responses of aggressive and non-aggressive ADHD children to methylphenidate (MPH) were noted in behavioral and laboratory measures [11,12].

Based on the current Diagnostic and Statistical Manual, Fourth Edition (DSM-IV) [3], ADHD is categorized into 3 subtypes, including inattentive type, hyperactive-impulsive type, and combined type, according to the predominant clinical manifestations of inattention, hyperactivity, and impulsivity. The validity of DSM-IV ADHD predominantly inattentive and combined types has been debated for decades [13,14]. Besides the clinical manifestations, differences in externalizing problems and impairments in school work and peer-related activity between subtypes have also been reported [15]. The effects of methylphenidate on the neuropsychological profiles of subtypes of ADHD patients are still controversial [16,17].

Of the stimulant medications, MPH is the most widely used in the pharmacological management of children with ADHD [18]. Some studies have demonstrated the acute neuropsychological effects of MPH in ADHD patients [19,20]. Acute MPH quickens response on a reaction time task and enhanced performance on some aspects of non-executive functioning [20]. It has also been noted that MPH increased the time spent on-task, and reduced time spent in distracted, impulsive, and random response states [19]. The temporal and sustaining effects on ADHD behavioral symptoms were significant after 4 months of MPH treatment [21]. The famous NIMH multi-model treatment study of ADHD (MTA) reported significant differences in hyperactive-impulsive symptoms with medication treatment within 14 months [22]. However, relatively few studies have investigated the long-term course of improvements in ADHD symptoms and their neurocognitive function; as well as the differences between ADHD subtypes.

Therefore, the aims of the present study were to determine: First, whether there are sustainable improvements in ADHD clinical symptoms and neurocognitive function in realistic clinical settings. Second, whether the differences in ADHD clinical symptoms and neurocognitive functions between ADHD subtypes exist along with the course of treatment. And, whether there were differences in improvements in ADHD symptoms between different classifications of ADHD patients.

## Methods

### Study participants

This observational-prospective study was conducted at the Child and Adolescent Psychiatry Out-patient Department of Chang Gung Memorial Hospital, Keelung, from May 2008 to June 2009. The study was approved by the Institutional Review Board (IRB) of Chang Gung Memorial Hospital. We recruited patients aged between 6 and 12 years old who met the criteria for ADHD outlined in the DSM-IV [3]. The diagnosis was made by a child psychiatrist in a structured interview using the Kiddie Epidemiologic Version of the Schedule for Affective Disorders and Schizophrenia (K-SADS-E) [23]. Development of the Chinese K-SADS-E was completed by the Child Psychiatry Research Group in Taiwan [24]. Patients were examined by child psychiatrists and excluded from the study if they had a history of major physical or psychiatric disease (such as pervasive developmental disorder, bipolar disorder, major depression, anxiety disorder, or psychosis), a history of substance abuse, or mental retardation. Included patients were either newly diagnosed with ADHD or had an existing diagnosis but had not taken medication for ADHD during the previous 6 months or more.

### Clinical Measures

Patients were interviewed by a clinician using the ADHD Rating Scale (ADHD-RS) and a computerized Continuous Performance Test (CPT). The social and behavioral competence and ADHD symptoms of patients were evaluated with the Child Behavior Checklist (CBCL), Swanson, Nolan, and Pelham, and Version IV Scale for ADHD (SNAP-IV), which the parents of the patients completed.

#### The Child Behavior Checklist (CBCL)

is a questionnaire completed by parents and teachers that evaluates the social and behavioral competence in the past 6 months of children aged between 4 and 16 years old [25,26]. The CBCL contains eight subscales: depression/anxiety, thought/obsessive, somatic complaint, social withdrawal, hyperactivity, aggressive behavior, delinquency, internalizing behavior, and externalizing behavior. A T-score of 50 for each scale indicates average functioning in reference to other children of the same age and gender, and every 10 points represents one standard deviation [25,27].

#### The ADHD-Rating Scale-parent (ADHD-RS)

by DuPaul et al. (1998) is a validated instrument with which clinicians assign ratings on the basis of information from the parent(s) and child [28]. It is an 18-item checklist derived from the 18 criteria outlined in the DSM-IV for diagnosing ADHD. Each of the items has a 4-point Likert scale scoring from 0 to 3 points (0 = never or rarely, 1 = sometimes, 2 = often and 3 = very often). ADHD-RS provides a total score (the sum of all 18 items), and can also be divided into inattentive (odd numbered items) and hyperactive/impulsive subscales (even numbered items). Higher scores indicate a greater severity of ADHD. The scale is reported to have good inter-rater reliability [29].

#### The Swanson, Nolan, and Pelham, and Version IV Scale (SNAP-IV)

is a 26-item questionnaire in a 4-point Likert scale that is used to evaluate ADHD symptoms and severity, and it is completed by parents and teachers [30,31]. The 26 items include 18 for ADHD symptoms (9 for inattentive, 9 for hyperactive/impulsive) and 8 for oppositional defiant disorder (ODD) symptoms as defined in the DSM-IV. Each item is scored on a 0-3 scale similar to the ADHD-RS (0 = not at all, 1 = just a little, 2 = quite a bit and 3 = very much). The SNAP-IV consists of Inattention, Hyperactivity/Impulsivity, and Oppositional subscales [30,31]. The Chinese version of the SNAP-IV was reported to have satisfactory levels of reliability and concurrent validity [32].

#### Continuous Performance Tests (CPT)

The computerized CPT involves the presentation of target and non-target stimuli. The test runs for 14 minutes and primarily assesses attention and impulse control [33,34]. Briefly, participants are required to respond to the stimuli on a computer screen by pressing a space bar for every letter except for the letter "X." Multiple dependent measures exist, including Omissions, Commissions, Response Time, Variability of Standard Error, and Detectability (D'). The Confidence Index (percentile) integrates all CPT data obtained to provide a chance out of 100 that a significant attention problem exists [33,34]. In terms of the reliability of Conners' CPT II, the pill-half reliability is 0.66-0.95, and test-retest reliability after 3 months is 0.55-0.84 [35].

### Study Procedure

This investigation comprised a 24-week, non-randomized, observational, prospective study. At visit 1 (baseline), each ADHD patient performed the CPT at around 9:00 AM; this took place in a room dedicated to testing so that test condition variability was minimized. The CBCL and SNAP-IV were completed by the patients' parents, and ADHD-RS ratings were made by a child psychiatrist. At visit 2 (one month from baseline), patients performed the CPT at 9:00 AM, around 1 to 2 hours after they had taken MPH. The SNAP-IV was completed by the patients' parents, and ADHD-RS ratings were made by the same rater. At visit 3 (3 months from baseline) and visit 4 (6 months from baseline), the same procedure as visit 2 was repeated.

Subjects were prescribed MPH at a dose range of 5 to 15 mg/day at visit 1 (V1), based on the severity of their clinical symptoms, and their age, height and body weight. Other concomitant medications were not allowed. Patients were advised to take MPH at least on weekday, but drug holiday was permitted. We confirmed the drug compliance at each visit according to the reports of patients' parents and the remnant drug. To ensure the study reflected real-life clinical practice, patient care was performed at the discretion of the psychiatrist. Modification of the MPH dose could take place at visit 2 (V2), visit 3 (V3) or visit 4 (V4). No treatment instructions were given other than that the psychiatrist should manage the subjects per their usual practice. Follow-up of the subjects was not limited by the study's schedule of assessments.

### Statistical Analyses

The data were analyzed with the statistical software package SPSS, Version 16. Variables are presented as either mean ± standard deviation (SD) or frequency.

The ADHD patients were divided into DSM-IV subtypes (Inattentive type, Hyperactive-impulsive type, and Combined type), and were also categorized into aggressive and non-aggressive subtypes, based on the aggression scale of the CBCL, with a cutoff point of 60. Chi-square was used to compare the rate of lost to follow-up between subtypes. The Student's *t*-test or One-way analysis of variance (ANOVA) was used to compare the demographic data and the CBCL, SNAP-IV, ADHD-RS, and CPT scores between ADHD subtypes.

Patients with a baseline assessment and at least 1 follow-up assessment were included in the efficacy analyses. Missing data were accounted for using the method of last observation carried forward (LOCF). The ADHD measures, except oppositional scores of SNAP-IV, were reduced by means of a principal components analysis (PCA) with a set of weights for a composite ADHD score for each subject. The composite score for each factor and oppositional scores of SNAP-IV were applied to the analysis for repeated-measure analysis of variance (ANOVA), followed by a post-hoc Least Significant Difference (LSD) test. We investigated the extent of the differences in changes of these composite ADHD scores for each factor between ADHD subtypes, also by repeated-measure analysis of variance (ANOVA), using average MPH dosage/body weight during 6 month as a covariate. The hypothesis that there is a differential change over 6 months would be supported by significant subtype × visit interactions on dependent measures. The factors which showed a significant interaction between subtypes and visits were taken into further analyzed. The changes from the baseline to the endpoint of these factors were computed, and the Student's *t*-test was applied for examine the significant differences of these changes between subtypes.

Two-tailed p values < 0.05 were considered statistically significant.

## Results

There were 50 ADHD patients (40 boys and 10 girls) with a mean age of 7.84 ± 1.64 years. Fifteen of them were inattentive type, 11 were hyperactive-impulsive type, and 24 were combined type. Using a cutoff point of 60 on the aggression scale of the CBCL, patients were also categorized into aggressive and non-aggressive subtypes: 28 into the aggressive subtype and 22 into the non-aggressive subtype. There was no significant difference in the categorization rates of patients with aggression between the DSM-IV subtypes. Table 1 presents and compares the demographic data and ADHD symptom measurements of the CBCL, SNAP-IV, ADHD-RS and CPT between DSM-IV subtypes at baseline, and which between aggressive and non-aggressive patients at baseline are displayed in Table 2.

**Table 1:** Demographic data and ADHD symptoms measurements for ADHD patients with DSM-IV subtypes at baseline

	Inattentive type (N = 15)	Hyperactive- impulsive type (N = 11)	Combined type (N = 24)	Test statistic	*P *value
**Gender**	**n(%)**	**n(%)**	**n(%)**		0.282
Male	10 (66.7)	9 (81.8)	21 (87.5)	2.53	
Female	5 (33.3)	2 (18.2)	3 (12.5)		
	**Mean(SD)**	**Mean(SD)**	**Mean(SD)**		
**Age **(years)	8.1 (1.8)	7.5 (1.6)	7.3 (1.5)	1.13	0.332
**Height **(cm)	130.5 (10.9)	127.8 (9.7)	126.5 (8.4)	0.82	0.445
**Weight **(kg)	29.5 (7.5)	27.7 (7.0)	26.7 (6.2)	0.77	0.467
**CBCL**					
Hyperactive	69.0 (12.4)	61.6 (8.2)	66.7 (8.3)	1.94	0.155
Aggression	63.3 (10.3)	64.6 (7.4)	59.3 (8.6)	1.69	0.196
Delinquency	65. 7(11.9)	61.1 (9.8)	61.8 (10.9)	0.74	0.485
**SNAP-IV**					
Inattentive	17.3 (3.8)	14.9 (4.4)	17.5 (4.2)	1.60	0.212
Hyperactive^a^	13.3 (5.8)	17.5 (4.7)	17.5 (4.6)	3.77	**0.030**
Oppositional	11.3 (5.8)	12.0 (4.8)	9.6 (4.4)	1.06	0.355
**ADHD-RS**					
Total score	30.8 (4.5)	31.8 (5.4)	34.3 (5.7)	2.17	0.125
Inattention^b^	16.5 (2.3)	12.7 (3.1)	17.9 (3.3)	11.11	**<0.001**
Hyperactivity^c^	14.3 (3.8)	19.2 (3.2)	16.4 (4.2)	4.88	**0.012**
**CPT**					
Confidence Index	53.7 (21.6)	58.3 (16.5)	63.4 (24.0)	0.91	0.408
Omission	53.4 (10.3)	53.3 (10.7)	63.7 (30.4)	1.32	0.277
Commission	46.4 (10.5)	47.4 (12.7)	48.3 (10.8)	0.13	0.877
Hit RT	57.6 (13.3)	52.9 (9.9)	58.7 (15.2)	0.70	0.502
Hit RT SE	54.3 (10.4)	54.8 (8.8)	60.5 (12.6)	1.78	0.181
Variability	53.1 (8.9)	54.4 (8.9)	58.4 (10.7)	1.52	0.230
Detectability	47.9 (8.7)	50.4 (13.2)	51.0 (8.2)	0.48	0.623
Response Style	50.7 (11.7)	47.5 (6.8)	54.0 (12.0)	1.39	0.258

CBCL = The Child Behavior Checklist; SNAP-IV = the Swanson, Nolan, and Pelham, and Version IV Scale; ADHD-RS = ADHD Rating Scale; CPT = The Computerized Continuous Performance Test; RT = reaction time; SE = Standard Error.

^a ^H > I, C > I, H≈C; ^b ^I > H, C > H, I≈C; ^c ^H > I, C≈H, I≈C

**Table 2:** Demographic data and ADHD symptoms measurements for ADHD patients with aggression and without aggressive at baseline

	Aggressive (N = 28)	Non-aggressive (N = 22)	Test statistic	*P *value
**Gender**	**n (%)**	**n (%)**		0.263
Male	21 (75.0)	19 (86.4)	0.48	
Female	7 (25.0)	3 (13.6)		
**ADHD subtype**			2.35	0.308
Inattentive	9 (32.1)	6 (27.3)		
Hyperactive-impulsive	8 (28.6)	3 (13.6)		
Combined	11 (39.3)	13 (59.1)		
	**Mean (SD)**	**Mean (SD)**		
**Age **(years)	7.6 (1.8)	7.6 (1.4)	0.06	0.955
**Height **(cm)	128.6 (8.6)	127.1 (10.6)	0.54	0.595
**Weight **(kg)	27.5 (5.7)	28.1 (8.0)	-0.29	0.776
**CBCL**				
Hyperactive	69.8 (8.8)	61.7 (9.3)	3.12	**0.003**
Aggression	68.1 (6.0)	53.6 (4.7)	9.42	**< 0.001**
Delinquency	69.8 (8.0)	54.0 (7.0)	7.31	**< 0.001**
**SNAP-IV**				
Inattentive	17.4 (4.8)	16.2 (3.3)	1.05	0.301
Hyperactive	17.7 (4.5)	14.4 (5.7)	2.32	**0.025**
Oppositional	12.9 (4.2)	7.8 (4.3)	4.24	**< 0.001**
**ADHD-RS**				
Total score	33.5 (6.0)	31.7 (4.6)	1.12	0.268
Inattention	15.7 (3.3)	17.1 (3.8)	-1.41	0.165
Hyperactivity	17.8 (4.0)	14.6 (3.8)	2.83	**0.007**
**CPT**				
Confidence Index	57.3 (21.1)	62.0 (22.9)	-0.76	0.450
Omission	57.5 (20.2)	59.3 (26.0)	-0.27	0.786
Commission	47.8 (11.6)	47.2 (10.2)	0.19	0.851
Hit RT	53.8 (11.1)	61.3 (15.4)	-1.98	0.053
Hit RT SE	54.8 (10.7)	60.6 (11.7)	-1.84	0.072
Variability	54.6 (10.3)	57.7 (9.2)	-1.11	0.275
Detectability	49.3 (10.1)	50.7 (9.0)	-0.53	0.598
Response Style	49.0 (6.9)	54.9 (14.4)	-1.89	0.065

CBCL = The Child Behavior Checklist; SNAP-IV = the Swanson, Nolan, and Pelham, and Version IV Scale; ADHD-RS = ADHD Rating Scale; CPT = The Computerized Continuous Performance Test; RT = reaction time; SE = Standard Error.

Among the 50 ADHD patients at the initial visit, 42, 33, and 30 patients remained in the study at visit 2, 3, and 4, respectively. The reasons for premature discontinuation were adverse events (N = 3), non-compliance (N = 4), withdrawal of consent (N = 2), and lost to follow-up (N = 11). There were no significant differences in discontinuation rates between DSM-IV subtypes ADHD patients (p = 0.905), or between aggressive and non-aggressive patients (p = 0.606). All patients were drug-free at visit 1 (baseline). The mean dose of MPH was 9.87 ± 5.09 mg (0.37 ± 0.20 mg/kg) at visit 2, 14.88 ± 6.97 mg (0.48 ± 0.29 mg/kg) at visit 3, and 13.00 ± 7.52 mg (0.46 ± 0.24 mg/kg) at visit 4, respectively.

To condense the number of ADHD measures and reduce type I errors, a principal components analysis was performed. Four factors yielding eigenvalues greater than 1.00 were retained for varimax rotation. The weights for the measures of each factor are listed in Table 3. The resultant factors were labeled on the basis of their clinical meaning: CPT distraction (factor 1), CPT impulsivity (factor 2), clinical hyperactivity (factor 3), and clinical inattention (factor 4). These 4 factors had eigenvalues of 3.99, 2.21, 1.44, and 1.16, respectively, and accounted for 79.93% of the total matrix variance.

**Table 3:** The structure of factors produced by principal components analysis of ADHD measures^a,^^b^.

	Factor 1 (CPT distraction)	Factor 2 (CPT impulsivity)	Factor 3 (Clinical hyperactivity)	Factor 4 (Clinical inattention)
**SNAP-IV**				
Inattention	0.08	0.07	0.36	**0.79**
Hyperactivity	0.09	0.08	**0.85**	0.29
**ADHD-RS**				
Inattention	0.19	0.11	-0.06	**0.89**
Hyperactivity	0.18	0.06	**0.93**	-0.04
**CPT**				
Omission	0.73	-0.06	0.19	0.34
Commission	-0.14	**0.96**	0.05	0.04
Hit RT	**0.76**	-0.43	-0.06	0.11
Hit RT SE	**0.93**	0.08	0.11	0.15
Variability	**0.84**	0.25	0.20	0.12
Detectability	0.21	**0.90**	0.09	0.16
Response style	**0.68**	0.02	0.03	-0.06

^a^Rotation Method: Varimax with Kaiser Normalization.^b^Absolute value of factor loadings greater than 0.50 for each variable in bold face type.

CPT = The Computerized Continuous Performance Test; SNAP-IV = the Swanson, Nolan, and Pelham, and Version IV Scale; ADHD-RS = ADHD Rating Scale; RT = reaction time; SE = Standard Error.

During the 6-month treatment, there were significant improvements in CPT impulsivity (F = 17.22, p < 0.001), clinical hyperactivity (F = 19.85, p < 0.001), and clinical inattention (F = 26.06, p < 0.001). However, CPT distraction was not improved (F = 0.80, p = 0.497), and there were no significant differences between any paired visits. For the rest three factors aforementioned, the trends of changes were the same during 6 months. There were significant improvements from V1 to V2, and V2 to V3, but no significant differences from V3 to V4. The oppositional scores of SNAP-IV significantly changed over 6 months (F = 22.74, p < 0.001), and there were significant differences from V1 to V2, and V3 to V4.

**Figure 1:**
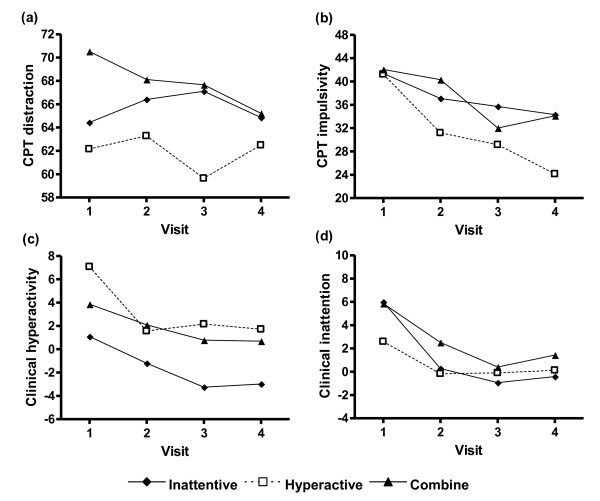
**Changes in ADHD symptom composite scores between DSM-IV subtypes of ADHD patients during 6 months of real-world clinical treatment.** There were no significant differences between DSM-IV subtypes in CPT distraction (a), CPT impulsivity (b), and clinical inattention (d).  For clinical hyperactivity (c), there was significant difference (F = 4.11, p = 0.024) between subtypes (H>I, C>I, H˜C). There were no significant interactions between DSM-IV subtypes and visits in these four composite scores.  I = inattentive type; H = hyperactive-impulsive type; C = combined type

In terms of the differences between DSM-IV subtypes, Figure 1 summarizes the results of changes over time for each of the four dependent factors. For CPT distraction, CPT impulsivity, and clinical inattention, there was no significant difference between the subtypes and no significant interaction between subtypes and visits in these factors. For clinical hyperactivity, there was significant difference (F = 4.11, p = 0.024) between subtypes, but no significant interactions between DSM-IV subtypes and visits.

**Figure 2:**
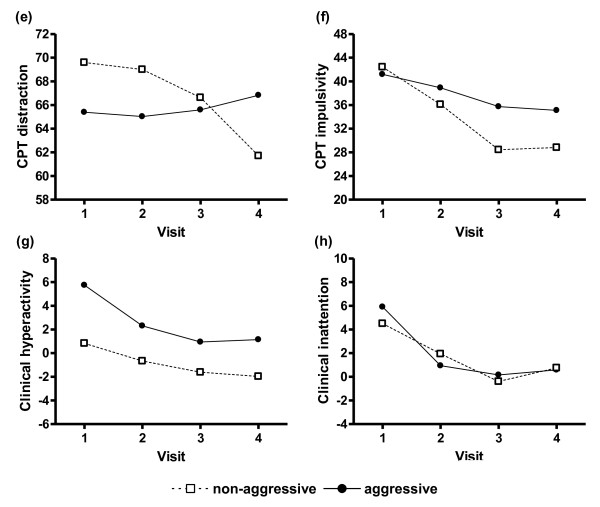
**Changes in ADHD symptom composite scores between aggressive and non-aggressive ADHD patients during 6 months of real-world clinical treatment**. There was a significant difference in clinical hyperactivity (g) between aggressive subtypes (F = 7.87, p = 0.008). There were significant interactions between aggressive subtypes and visits in CPT distraction (e) (F = 3.05, p = 0.031) and CPT impulsivity (f) (F = 3.53, p = 0.017). There was neither significant difference in clinical inattention (h) between subtypes, nor interactions between subtypes and visits in this factor.

For the differences between aggressive and non-aggressive patients, the results were more diverse in each factor. Figure 2 demonstrate the results of changes over time for each of the four dependent factors. For CPT distraction, there was no significant difference between subtypes, but there was significant interaction between subtypes and visits (F = 3.05, p = 0.031). The changes from V1 to V4 in non-aggressive patients were significantly greater than aggressive patients (t = 2.27, p = 0.028). Similarly for CPT impulsivity, there was no significant difference between subtypes, but there was also significant interaction between subtypes and visits (F = 3.53, p = 0.017). The changes from V1 to V4 in non-aggressive patients were significantly greater than aggressive patients (t = 2.39, p = 0.021). For clinical hyperactivity, there was a significant difference between subtypes (F = 7.87, p = 0.008), but no significant interaction between subtypes and visits. For clinical inattention, there were neither significant differences between subtypes nor an interaction between subtypes and visits.

## Discussion

The results of our study showed significant improvements in clinical hyperactivity, inattention, and CPT impulsivity composited scores, but not in CPT distraction scores during the 6 months of real-world clinical treatment. There were significant differences in clinical hyperactivity between ADHD patients sub-grouping both by DSM-IV subtype and by CBCL aggressive scale. There were no interactions between DSM-IV subtypes and visits in these 4 dimensions of clinical symptoms and cognitive performance among ADHD patients. Nevertheless, the interactions between sub-grouping by CBCL aggressive scale and visits were significant in the CPT performance.

Optimal performance on the CPT is achieved by responding quickly and not making mistakes [36]. Some studies suggested that CPT performance measures appeared to be highly correlated to the constellation of ADHD symptoms [37]. In the traditional understanding of how CPT results relate to ADHD behaviors, errors of commission and omission are assumed to reflect impulsivity and symptoms of inattention, respectively [37,38]. In general, CPT is a relatively objective index which showed less placebo effects and rating bias [39]. CPT performances of children with ADHD significantly improve after a single dose of MPH [19]. We suggest that the CPT impulsivity response improved along with clinical ADHD symptoms under 6-month realistic clinical setting, but the distraction response did not.

Differences in the neuropsychological profiles and effects of MPH between DSM-IV subtyping ADHD patients have been reported [16,17]. Chhabildas et al. (2001) showed similar profiles of impairment on neuropsychological measures in hyperactive and non-hyperactive patients [16]. Gorman et al. demonstrated that MPH ameliorated task-incompatible behavior and attention comparably in both ADHD subtypes, but hyperactivity and aggression were reduced largely in hyperactive types [17]. ADHD subtypes differed along with symptom severity in childhood, but these differences were no longer significant in adolescents [40]. In our study, there were no interactions of DSM-IV subtypes with these 4 dimensions of clinical symptoms and cognitive performance among ADHD patients. The discriminating validity for the effectiveness of MPH by ADHD sub-grouping by DSM-IV was not supported.

Different characteristics of behavioral and neurocognitive performance were investigated between aggressive and non-aggressive ADHD patients, and classified by the IOWA Conners or CBCL [11,12,41]. Klorman et al. reported improvement in ADHD behavior and accuracy and speed on the CPT for both groups under MPH treatment [41]. Barkley et al. demonstrated a similar drug response in these two groups, however, the non-aggressive patients had linear decrease in error rates of CPT commission parameter [11]. Matier et al. reported that both ADHD groups had a significant decrease in attention, but the activity level decreased only in the non-aggressive ADHD group, after medication [12]. In our study, non-aggressive ADHD patients showed a greater degree of improvement in CPT performance from medication than aggressive ones. These results might indicate that non-aggressive ADHD patients had a higher potential for improving in neurocognitive function than aggressive ones.

These results also supported the hypothesis that aggressive and non-aggressive ADHD patients might have different underlying determinants. Diminished central serotonergic (5-HT) activity has been linked to impulsivity and aggression [42]. Catecholaminergic (CA) mechanisms have been more strongly implicated as neurobiological factors of ADHD, especially in the dopaminergic system [43]. The mechanism of MPH which inhibits the reuptake of dopamine increases synaptic dopamine and dopaminergic neurotransmission [44]. Thus, the primary effect of MPH on central CA mechanisms might have a significantly greater impact on the non-aggressive ADHD patient, whose deficits are hypothesized to be mediated by CA. The aggressive ADHD patient, whose deficits are hypothesized to be partly related to 5-HT mechanisms, had less response to MPH [12].

The course of ADHD symptom improvement has been demonstrated in some studies. However, most of these studies focused on the acute effects of MPH on neuropsychological tests [19,20]. Other studies that investigated the sustainable effects of MPH on ADHD behavioral symptoms often simply compared the endpoint with the baseline of the studies [21,22]. In our study, we provided the time course of ADHD symptom improvement with MPH treatment in the real world setting. The hyperactive-impulsive, inattentive and oppositional ADHD clinical symptoms were significantly improved during 6 months. The results are generally identical to the previous literature [22], as well as clinical experience of many child psychiatrists.

### Limitations

Some limitations of this study need to be considered. First, this was an open labeled, non-randomized study, so the placebo effects, rating bias, and reporting bias could not be ruled out. In addition, there was no data of ADHD patients on placebo or non-medicated for comparison; hence, we could not certainly justify these results derived from effects of MPH or time. Second, the aggressive and non-aggressive ADHD patients were roughly divided by the aggressive behavior scales of the CBCL with a cutoff point of 60. A suitable index has been suggested for two standard deviations above the normal mean on the Aggressive scale (T score > 70) [11]. However, the clinically useful cutoff point of 60, instead of 70, was effective in discriminating between ADHD patients with and without comorbid diagnoses [45]. Racial and ethnic differences in psychopathology and symptom severity have been reported [46]. Only 8 subjects in our study had a T score above 70 on the aggressive scale of the CBCL. Thus, we finally chose a cutoff point of 60 on the aggressive scale to subdivide the ADHD patients into aggressive and non-aggressive subtypes. Furthermore, the correlation of aggression and hyperactivity might hinder the distinguishability in predicting outcome, so there might be a more valid way to make subgroups. Third, the treatment procedure was not standardized, so there was a possible confounding effect from the MPH dosage, although MPH doses/body weight was used as a covariate in the analyses. Finally, the sample size of our study was not sufficiently large, so the study might not have adequate statistical power to detect possible differences in ADHD symptoms and CPT performance between ADHD subtypes. Meanwhile, the dropout rate may have reduced the statistical power and influenced the results. Caution should be taken in applying the results to clinical practice.

## Conclusions

While having limitations, our study has strengths relative to studies on similar topics. First, we used longitudinal evidence of changes in ADHD symptoms, rather than a cross-sectional observation or an acute response to MPH. Second, we measured many dimensions of ADHD symptomatology, with scores derived from information provided by the patients' parents (SNAP-IV) and clinical observers (ADHD-RS), and from performance on a neurocognitive test (CPT). Last, we used different categories in comparing ADHD symptom improvements.

We suggest ADHD symptoms, which include impulsivity performance in the CPT and clinical inattention and hyperactivity dimensions, were significantly improved during 6 months in realistic clinical settings. The non-aggressive ADHD patients might have a higher potential for improving in CPT performance than aggressive ones. However, it warrant further investigation whether the different classifications of ADHD patients could be valid for predicting the improvements in ADHD patients' clinical symptoms and neurocognitive performance.

## Competing interests

The authors declare that they have no competing interests.

## Authors' contributions

LJW, YSH and YLC conceived the study, recruited the participants, and wrote the paper. CCH and ZYS gathered and analyzed the data. CKC carried out the literature search and helped to draft the manuscript. All authors read and approved the final manuscript.

## Pre-publication history

The pre-publication history for this paper can be accessed here:

http://www.biomedcentral.com/1471-244X/11/65/prepub

## References

[B1] Goldman LS Genel M Bezman RJ Slanetz PJ Diagnosis and treatment of attention-deficit/hyperactivity disorder in children and adolescents. Council on Scientific Affairs, American Medical Association JAMA 1998 279 1100 1107 10.1001/jama.279.14.1100 9546570

[B2] Gau SS Chong MY Chen TH Cheng AT A 3-year panel study of mental disorders among adolescents in Taiwan Am J Psychiatry 2005 162 1344 1350 10.1176/appi.ajp.162.7.1344 15994718

[B3] American Psychiatric Association American Psychiatric Association. Diagnostic and Statistical Manual of Mental disorders In 4 (ed), DSM-IV-TR (Text Revision) 2000 Washington, DC

[B4] Spencer TJ Biederman J Mick E Attention-deficit/hyperactivity disorder: diagnosis, lifespan, comorbidities, and neurobiology J Pediatr Psychol 2007 32 631 642 10.1093/jpepsy/jsm005 17556405

[B5] Losier BJ McGrath PJ Klein RM Error patterns on the continuous performance test in non-medicated and medicated samples of children with and without ADHD: a meta-analytic review J Child Psychol Psychiatry 1996 37 971 987 10.1111/j.1469-7610.1996.tb01494.x 9119944

[B6] Rubia K Taylor E Smith AB Oksanen H Overmeyer S Newman S Neuropsychological analyses of impulsiveness in childhood hyperactivity Br J Psychiatry 2001 179 138 143 10.1192/bjp.179.2.138 11483475

[B7] Hinshaw SP On the distinction between attentional deficits/hyperactivity and conduct problems/aggression in child psychopathology Psychol Bull 1987 101 443 463 3602250

[B8] Milich R Loney J Landau S Independent dimensions of hyperactivity and aggression: a validation with playroom observation data J Abnorm Psychol 1982 91 183 198 7096789 10.1037//0021-843x.91.3.183

[B9] Kryzhanovskii GN Makul'kin RF Shandra AA Role of hyperactive determinant structures in the creation of functional complexes of seizure activity in the cerebral cortex Neurosci Behav Physiol 1979 9 401 405 492510 10.1007/BF01185065

[B10] Gittelman R Mannuzza S Shenker R Bonagura N Hyperactive boys almost grown up. I. Psychiatric status Arch Gen Psychiatry 1985 42 937 947 4037987 10.1001/archpsyc.1985.01790330017002

[B11] Barkley RA McMurray MB Edelbrock CS Robbins K The response of aggressive and nonaggressive ADHD children to two doses of methylphenidate J Am Acad Child Adolesc Psychiatry 1989 28 873 881 10.1097/00004583-198911000-00011 2808257

[B12] Matier K Halperin JM Sharma V Newcorn JH Sathaye N Methylphenidate response in aggressive and nonaggressive ADHD children: distinctions on laboratory measures of symptoms J Am Acad Child Adolesc Psychiatry 1992 31 219 225 10.1097/00004583-199203000-00007 1564022

[B13] Morgan AE Hynd GW Riccio CA Hall J Validity of DSM-IV ADHD predominantly inattentive and combined types: relationship to previous DSM diagnoses/subtype differences J Am Acad Child Adolesc Psychiatry 1996 35 325 333 10.1097/00004583-199603000-00014 8714321

[B14] Milich R Balentine AC Lynam DR ADHD Combined Type and ADHD Predominantly Inattentive Type Are Distinct and Unrelated Disorders Clinical Psychology: Science and Practice 2001 8 463 488 10.1093/clipsy/8.4.463

[B15] Graetz BW Sawyer MG Hazell PL Arney F Baghurst P Validity of DSM-IV ADHD subtypes in a nationally representative sample of Australian children and adolescents Journal of the American Academy of Child and Adolescent Psychiatry 2001 40 1410 1417 10.1097/00004583-200112000-00011 11765286

[B16] Chhabildas N Pennington BF Willcutt EG A comparison of the neuropsychological profiles of the DSM-IV subtypes of ADHD J Abnorm Child Psychol 2001 29 529 540 10.1023/A:1012281226028 11761286

[B17] Gorman EB Klorman R Thatcher JE Borgstedt AD Effects of methylphenidate on subtypes of attention-deficit/hyperactivity disorder J Am Acad Child Adolesc Psychiatry 2006 45 808 816 10.1097/01.chi.0000214191.57993.dd 16832317

[B18] Swanson JM Gupta S Williams L Agler D Lerner M Wigal S Efficacy of a new pattern of delivery of methylphenidate for the treatment of ADHD: effects on activity level in the classroom and on the playground J Am Acad Child Adolesc Psychiatry 2002 41 1306 1314 10.1097/00004583-200211000-00011 12410072

[B19] Teicher MH Lowen SB Polcari A Foley M McGreenery CE Novel strategy for the analysis of CPT data provides new insight into the effects of methylphenidate on attentional states in children with ADHD J Child Adolesc Psychopharmacol 2004 14 219 232 10.1089/1044546041648995 15319019

[B20] Rhodes SM Coghill DR Matthews K Acute neuropsychological effects of methylphenidate in stimulant drug-naive boys with ADHD II--broader executive and non-executive domains J Child Psychol Psychiatry 2006 47 1184 1194 10.1111/j.1469-7610.2006.01633.x 17076758

[B21] Schachar RJ Tannock R Cunningham C Corkum PV Behavioral, situational, and temporal effects of treatment of ADHD with methylphenidate J Am Acad Child Adolesc Psychiatry 1997 36 754 763 10.1097/00004583-199706000-00011 9183129

[B22] The MTA Cooperative Group A 14-month randomized clinical trial of treatment strategies for attention-deficit/hyperactivity disorder. The MTA Cooperative Group. Multimodal Treatment Study of Children with ADHD Arch Gen Psychiatry 1999 56 1073 1086 10.1001/archpsyc.56.12.1073 10591283

[B23] Kaufman J Birmaher B Brent D Rao U Flynn C Moreci P Williamson D Ryan N Schedule for Affective Disorders and Schizophrenia for School-Age Children-Present and Lifetime Version (K-SADS-PL): initial reliability and validity data J Am Acad Child Adolesc Psychiatry 1997 36 980 988 10.1097/00004583-199707000-00021 9204677

[B24] Gau SF Soong WT Psychiatric comorbidity of adolescents with sleep terrors or sleepwalking: a case-control study Aust N Z J Psychiatry 1999 33 734 739 10544999 10.1080/j.1440-1614.1999.00610.x

[B25] Achenbach T ed Manual for the revised child Behavior Checklist 1991 Burlington: University of Vermont, Department of Psychiatry

[B26] Biederman J Monuteaux MC Kendrick E Klein KL Faraone SV The CBCL as a screen for psychiatric comorbidity in paediatric patients with ADHD Arch Dis Child 2005 90 1010 1015 10.1136/adc.2004.056937 16177156 PMC1720123

[B27] Kim JW Park KH Cheon KA Kim BN Cho SC Hong KE The child behavior checklist together with the ADHD rating scale can diagnose ADHD in Korean community-based samples Can J Psychiatry 2005 50 802 805 16408529 10.1177/070674370505001210

[B28] Zhang S Faries DE Vowles M Michelson D ADHD Rating Scale IV: psychometric properties from a multinational study as a clinician-administered instrument Int J Methods Psychiatr Res 2005 14 186 201 10.1002/mpr.7 16395872 PMC6878282

[B29] Gomez R Item response theory analyses of the parent and teacher ratings of the DSM-IV ADHD rating scale J Abnorm Child Psychol 2008 36 865 885 10.1007/s10802-008-9218-8 18266103

[B30] Swanson JM Kraemer HC Hinshaw SP Arnold LE Conners CK Abikoff HB Clevenger W Davies M Elliott GR Greenhill LL Hechtman L Hoza B Jensen PS March JS Newcorn JH Owens EB Pelham WE Schiller E Severe JB Simpson S Vitiello B Wells K Wigal T Wu M Clinical relevance of the primary findings of the MTA: success rates based on severity of ADHD and ODD symptoms at the end of treatment J Am Acad Child Adolesc Psychiatry 2001 40 168 179 10.1097/00004583-200102000-00011 11211365

[B31] Bussing R Fernandez M Harwood M Wei H Garvan CW Eyberg SM Swanson JM Parent and teacher SNAP-IV ratings of attention deficit hyperactivity disorder symptoms: psychometric properties and normative ratings from a school district sample Assessment 2008 15 317 328 10.1177/1073191107313888 18310593 PMC3623293

[B32] Liu YC Liu SK Shang CY Lin CH Tu CL Gau SF Norm of the Chinese Version of the Swanson, Nolan and Pelham, Version IV Scale for ADHD Taiwanese J Psychiatry 2006 20 290 304

[B33] Conners CK The computerized continuous performance test Psychopharmacol Bull 1985 21 891 892 4089110

[B34] Conners CK ed Conners' Continuous Performance Test II (CPTII) for Windows Technical Guide and Software Manual 2004 NY: MHS21510895

[B35] Chen KC Chu CL Yang YK Yeh TL Lee IH Chen PS Lu RB The relationship among insight, cognitive function of patients with schizophrenia and their relatives' perception Psychiatry Clin Neurosci 2005 59 657 660 10.1111/j.1440-1819.2005.01433.x 16401240

[B36] Pearson DA Santos CW Casat CD Lane DM Jerger SW Roache JD Loveland KA Lachar D Faria LP Payne CD Cleveland LA Treatment effects of methylphenidate on cognitive functioning in children with mental retardation and ADHD J Am Acad Child Adolesc Psychiatry 2004 43 677 685 10.1097/01.chi.0000124461.81324.13 15167084

[B37] Epstein JN Erkanli A Conners CK Klaric J Costello JE Angold A Relations between Continuous Performance Test performance measures and ADHD behaviors J Abnorm Child Psychol 2003 31 543 554 10.1023/A:1025405216339 14561061

[B38] Riccio CA Waldrop JJ Reynolds CR Lowe P Effects of stimulants on the continuous performance test (CPT): implications for CPT use and interpretation J Neuropsychiatry Clin Neurosci 2001 13 326 335 10.1176/appi.neuropsych.13.3.326 11514638

[B39] Madaan V Daughton J Lubberstedt B Mattai A Vaughan BS Kratochvil CJ Assessing the efficacy of treatments for ADHD: overview of methodological issues CNS Drugs 2008 22 275 290 10.2165/00023210-200822040-00002 18336058

[B40] Hurtig T Ebeling H Taanila A Miettunen J Smalley SL McGough JJ Loo SK Jarvelin MR Moilanen IK ADHD symptoms and subtypes: relationship between childhood and adolescent symptoms J Am Acad Child Adolesc Psychiatry 2007 46 1605 1613 10.1097/chi.0b013e318157517a 18030082

[B41] Klorman R Brumaghim JT Salzman LF Strauss J Borgstedt AD McBride MC Loeb S Effects of methylphenidate on attention-deficit hyperactivity disorder with and without aggressive/noncompliant features J Abnorm Psychol 1988 97 413 422 3060506 10.1037/0021-843X.97.4.413

[B42] Brown GL Ebert MH Goyer PF Jimerson DC Klein WJ Bunney WE Goodwin FK Aggression, suicide, and serotonin: relationships to CSF amine metabolites Am J Psychiatry 1982 139 741 746 6177256 10.1176/ajp.139.6.741

[B43] Zametkin AJ Rapoport JL Neurobiology of attention deficit disorder with hyperactivity: where have we come in 50 years? J Am Acad Child Adolesc Psychiatry 1987 26 676 686 10.1097/00004583-198709000-00011 2889717

[B44] Seeger G Schloss P Schmidt MH Marker gene polymorphisms in hyperkinetic disorder--predictors of clinical response to treatment with methylphenidate? Neurosci Lett 2001 313 45 48 10.1016/S0304-3940(01)02253-4 11684336

[B45] Biederman J Ball SW Monuteaux MC Kaiser R Faraone SV CBCL clinical scales discriminate ADHD youth with structured-interview derived diagnosis of oppositional defiant disorder (ODD) J Atten Disord 2008 12 76 82 10.1177/1087054707299404 17494835

[B46] Nguyen L Huang LN Arganza GF Liao Q The influence of race and ethnicity on psychiatric diagnoses and clinical characteristics of children and adolescents in children's services Cultur Divers Ethnic Minor Psychol 2007 13 18 25 17227173 10.1037/1099-9809.13.1.18

